# Role of ionotropic GABA, glutamate and glycine receptors in the tonic and reflex control of cardiac vagal outflow in the rat

**DOI:** 10.1186/1471-2202-11-128

**Published:** 2010-10-13

**Authors:** Cara M Hildreth, Ann K Goodchild

**Affiliations:** 1 Australian School of Advanced Medicine, Macquarie University, Sydney, Australia

## Abstract

**Background:**

Cardiac vagal preganglionic neurons (CVPN) are responsible for the tonic, reflex and respiratory modulation of heart rate (HR). Although CVPN receive GABAergic and glutamatergic inputs, likely involved in respiratory and reflex modulation of HR respectively, little else is known regarding the functions controlled by ionotropic inputs. Activation of g-protein coupled receptors (GPCR) alters these inputs, but the functional consequence is largely unknown. The present study aimed to delineate how ionotropic GABAergic, glycinergic and glutamatergic inputs contribute to the tonic and reflex control of HR and in particular determine which receptor subtypes were involved. Furthermore, we wished to establish how activation of the 5-HT_1A _GPCR affects tonic and reflex control of HR and what ionotropic interactions this might involve.

**Results:**

Microinjection of the GABA_A _antagonist picrotoxin into CVPN decreased HR but did not affect baroreflex bradycardia. The glycine antagonist strychnine did not alter HR or baroreflex bradycardia. Combined microinjection of the NMDA antagonist, MK801, and AMPA antagonist, CNQX, into CVPN evoked a small bradycardia and abolished baroreflex bradycardia. MK801 attenuated whereas CNQX abolished baroreceptor bradycardia. Control intravenous injections of the 5-HT_1A _agonist 8-OH-DPAT evoked a small bradycardia and potentiated baroreflex bradycardia. These effects were still observed following microinjection of picrotoxin but not strychnine into CVPN.

**Conclusions:**

We conclude that activation of GABA_A _receptors set the level of HR whereas AMPA to a greater extent than NMDA receptors elicit baroreflex changes in HR. Furthermore, activation of 5-HT_1A _receptors evokes bradycardia and enhances baroreflex changes in HR due to interactions with glycinergic neurons involving strychnine receptors. This study provides reference for future studies investigating how diseases alter neurochemical inputs to CVPN.

## Background

Cardiac vagal preganglionic neurons (CVPN) are found predominantly in the nucleus ambiguus (NA) as well as dorsal motor nucleus of the vagus (DMNV) and in the intermediate zone (IZ) between these two nuclei [1-3]. Activation of CVPN has negative chronotropic, dromotropic and ionotropic effects on the heart [4-6] and the activity of these neurons is increased in response to baroreceptor stimulation [7-9] and inhibited during inspiration [10,11].

Surprisingly little is known about the functional significance of inputs to CVPN mediated by either ionotropic or g-protein coupled receptors (GPCR). CVPN receive substantial inputs from ionotropic receptors. Microinjection of the GABA_A _receptor antagonist bicuculline into the NA evokes a profound decrease in HR [12] demonstrating that there is a large GABAergic input to CVPN that plays a role in setting the tonic level of heart rate (HR). GABAergic inputs also appear to mediate the inspiratory related inhibition of CVPN and may play a role in generating respiratory sinus arrhythmia (RSA) [13]. Although glycine evokes tachycardia when injected unilaterally into the NA [14], the role of glycinergic inputs in both the tonic and reflex control of cardiac vagal outflow have not been systematically explored. Glutamatergic inputs, conversely, mediate baroreceptor dependent excitation of CVPN [7,15-17], however the glutamate receptor subtype(s) involved has yet to be determined.

The influence some GPCR have in regulating CVPN has been determined. For example, activation of serotonin-1a/7, dopamine, μ-opioid or neurokinin-1 receptors within the NA decreases HR whereas activation of opioid-receptor like receptor-1 increases HR, demonstrating that activation of some GPCR can differentially alter the tonic level of HR [18-21]. The role of these GPCR, or for that matter others, however, in the reflex modulation of HR is not well understood. Systemic activation of serotonin-1A (5-HT_1A_) receptors potentiates baroreflex mediated bradycardia [22,23]. Central administration of the selective 5-HT_1A _antagonist, WAY-100635, attenuates baroreflex sensitivity suggesting a pivotal role for 5-HT_1A _receptors in the maintenance of reflex cardiac vagal outflow [24]. Furthermore, 5-HT_1A _receptor modulation of reflex cardiac vagal outflow is absent in the flinders sensitive line rat, an animal model of depression, which exhibits reduced BRS [23], thus highlighting the clinical importance of functional 5-HT_1A _receptor control of cardiac vagal outflow.

How activation of 5-HT1A receptors produces bradycardia and enhances baroreflex bradycardia is unknown. Speculation suggests that as the 5-HT_1A _receptor is an inhibitory GPCR, the mechanism must involve an interaction with inhibitory ionotropic inputs to CVPN [25]. In keeping with this hypothesis, application of 8-OH-DPAT *in vitro *attenuates presynaptic GABAergic and glycinergic inputs to CVPN [26,27]; however the functional significance of these inputs is unknown. Whether or not the effects of 5-HT_1A _activation on HR or BRS are mediated by GABAergic and/or glycinergic inhibition of CVPN in the ventrolateral medulla has not been investigated *in vivo*.

The initial aim of this study was to assess comprehensively, in the rat, the roles of GABA_A_, strychnine-sensitive glycine and NMDA and AMPA receptors within regions of the medulla containing CVPN in the tonic and reflex control of HR. Secondly, we investigated if activation of the 5-HT_1A _receptor evoked bradycardia and potentiation of baroreflex bradycardia was dependent upon GABAergic or glycinergic neurotransmission to CVPN.

## Methods

All experiments were approved by Macquarie University animal ethics committee and conducted in accordance with the Australian Code of Practice for the Care and Use of Animals for Scientific Purposes. Animals were housed on a 12 h light dark cycle (lights on at 6 am) with food and water available *ad libitum*.

Adult male Sprague Dawley (n = 34, 300-600 g) rats were anaesthetised with ethyl carbamate (urethane 1.3 g/kg ip; Sigma Aldrich Ltd.). Depth of anaesthesia was assessed regularly using reflex responses to tactile (corneal stroking) stimuli. Additional doses of urethane (0.13 g/kg IV) were administered as required. The right femoral artery and vein were cannulated to enable recording of arterial pressure (AP) and to administer drugs, respectively. A tracheotomy was performed. Rectal temperature was monitored and maintained at 37°C, using a thermostatically controlled electric blanket (Harvard Apparatus, USA) and an infrared-heating source. Animals were then placed in a stereotaxic frame and artificially ventilated with oxygen enriched room air. Peak expired CO_2 _was continuously monitored and maintained between 3.5-4.5%. A laminectomy was performed at C8, the spinal cord transected, and an occipital craniotomy was performed to expose the dorsal surface of the medulla.

### Experimental Protocol

#### Location of CVPN

In all animals, sites were located within the ventrolateral medulla from which vagally mediated bradycardia was evoked by 50 nl microinjections of L-glutamate (100 mM; Sigma Aldrich Ltd.). Microinjections of L-glutamate were made in a region encompassing 1.4-2.0 mm lateral to the midline, 0-1.2 mm rostral to the calamus scriptorius and between 1.5-3.5 mm ventral to the dorsal surface of the medulla. Tracks were separated by at least 0.3 mm and sites within a track by 0.5 mm or more. At each injection site the change in HR and/or presence of atrioventricular (AV) block, determined by the absence of QRS complex on the ECG waveform, were determined. The location of CVPN was identified as a site where a bradycardia > 50 bpm or AV block was evoked by microinjection of L-glutamate. In each experiment, 4 sites (2 per side) where the largest bradycardia or AV block concurrent with bradycardia were evoked, were targeted in order to cover the entire rostro-caudal extent of CVPN. At the end of each experiment these sites were marked with pontamine sky blue and the rat euthanased with potassium chloride (3 M). The medulla was removed and placed in 4% formaldehyde in phosphate buffer (0.1 M). Coronal sections (100 μm) were cut with a vibrating microtome and stained with cresyl violet for histological analysis.

#### Effect of spinal cord transection on basal cardiovascular parameters

In 7 animals the effect of spinal cord transection on HR, mean AP (MAP) and BRS was examined. MAP and HR were calculated over an 80 sec period immediately prior to spinal cord transection and after the peak fall in AP was reached and maintained at a steady level (roughly 30-45 mins post transection). Baroreceptor function curves were generated using bolus injections of phenylephrine (10 μg/kg in 0.1 ml saline IV) prior to spinal cord transection and after the peak fall in AP, and BRS was then calculated.

#### Drug microinjections

Twenty-five animals received drug microinjections into cardioinhibitory sites of the medulla. Once the two most cardiac responsive sites per side (i.e. four sites in total) were located, the drug to be tested (as indicated below) was microinjected into each of the sites (100 nl per injection, 2 injections per side) identified. The criteria for a cardiac responsive site were bradycardia exceeding 50 bpm or AV block and that the 4 sites covered the rostro-caudal extent of CVPN and were ~600 μm apart.

Animals either received two 100 nl bilateral microinjections of muscimol (GABA_A _agonist, 100 mM n = 4; Sigma-Aldrich Inc.), picrotoxin (GABA_A _receptor antagonist, 2 mM n = 6 [28]), bicuculline methiodide (GABA_A _antagonist, 0.4 mM n = 4 [29]; Sigma-Aldrich Inc.), strychnine hydrochloride (strychnine sensitive glycine receptor antagonist, 3 mM n = 4 [30]; Sigma-Aldrich Inc.), MK801 (NMDA receptor antagonist, 5 mM n = 4 [31]; Sigma-Aldrich Inc.) or CNQX (AMPA receptor antagonist, 2 mM n = 3 [32]; Research Biomedical International). Prior to drug microinjection and again at nadir cardiac response, baroreceptor function was assessed using changes in AP in response to bolus injections of phenylephrine (10 μg/kg in 0.1 ml IV). HR was calculated over three 80 second segments prior to drug microinjection and at the peak HR response. In rats receiving MK801 (n = 4), CNQX (2 mM) was then microinjected into the vicinity of CVPN following examination of baroreceptor function in order to examine effects of combined NMDA and AMPA receptor blockade on cardiac vagal function. In rats receiving microinjection of picrotoxin or strychnine, 8-OH-DPAT (0.1 mg/kg IV) was administered and baroreceptor curves generated again. In a separate cohort of rats, 8-OH-DPAT (0.1 mg/kg IV, n = 9) was administered and baroreceptor function curves generated at the peak cardiovascular response.

### Data Analysis

Data was acquired using CED1401 analogue-digital converter hardware and analysed off-line using Spike 2 software v. 6.2 (both from CED, Cambridge, UK).

#### BRS

The index method was used to calculate BRS. The index method calculates BRS as the ratio between the decrease in HR and increase in MAP (ΔHR/ΔMAP) induced by phenylephrine. BRS estimated using this method does not differ from BRS estimated using linear regression analysis [33]. BRS calculated from each replicate curve generated was averaged to give one final BRS estimate.

### Statistical Analysis

All data are presented as mean ± SEM. Paired or ratio t-tests were used to determine if each treatment affected HR or BRS. One-way ANOVA was used to compare effect of 8-OH-DPAT on HR under control conditions versus following picrotoxin or strychnine. *P *< 0.05 was considered significant (GraphPad Prism v5).

## Results

### Effect of lower cervical spinal cord transection on basal cardiovascular variables

In 7 animals, the effect of transection of the spinal cord at the 8th cervical segment on MAP, HR and baroreflex function was determined. Spinal cord transection evoked a profound fall in MAP (98.2 ± 4.2 v 60.2 ± 4.6 mmHg *P *< 0.001) and reduced resting HR (344 ± 15 v 302 ± 10 bpm *P *< 0.05). This hypotensive condition caused by reduced sympathetic activity also ensures that the baroreceptors are fully unloaded. Baroreflex mediated changes in HR, in response to induced increases in AP with phenylephrine, were unaffected by spinal cord transection and both the gain (0.76 ± 0.18 v 0.43 ± 0.10 bpm/mmHg *P *= 0.0955) and range (45 ± 10 v 37 ± 9 bpm *P *= 0.4637) of the baroreflex was not altered.

### Location and verification of CVPN sites in the medulla

**Figure 1:**
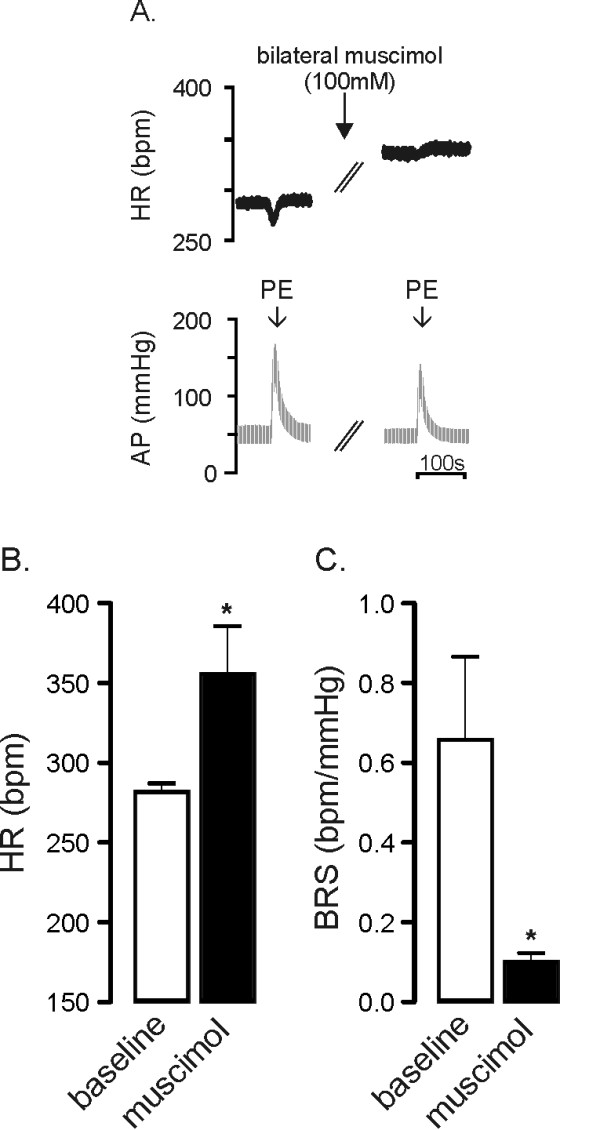
**Verification that dual bilateral microinjections in the ventral medulla inhibits cardiac vagal outflow**. A representative trace is provided in panel A showing that microinjection of muscimol (100 mM, 100 nl) into four regions of the medulla where bradycardia greater than 50 bpm was evoked by prior microinjection of L-glutamate (100 mM, 50 nl) increases resting heart rate (HR) and prevents baroreflex mediated bradycardia. Grouped data (n = 4) shows that microinjection of muscimol into these four sites evokes a large increase in HR (panel B) and dramatically reduces baroreflex sensitivity (BRS, panel C). * *P *< 0.05.

The medulla was mapped by glutamate microinjection to determine the sites from which a vagally mediated bradycardia could be evoked. Vehicle injection into these regions did not affect resting HR (340 ± 5 vs. 337 ± 10 bpm before vs. after vehicle injection, *P *= 0.6595 n = 3 and as previously described [19,20,34]). To verify that the sites targeted contained CVPN and that the entire rostro-caudal distribution of CVPN in the medulla could be targeted with two bilateral microinjections, bilateral microinjections of the GABA_A _agonist muscimol were made in 4 animals. The effects of muscimol on HR and baroreflex function are shown in Figure 1A. HR rose to 356 ± 15 bpm from 282 ± 3 bpm (*P *< 0.05, Figure 1B) and this was associated with a reduction in BRS (0.66 ± 0.21 bpm/mmHg v 0.10 ± 0.02 bpm/mmHg, *P *< 0.05, Figure 1C), effectively abolishing baroreflex control of HR. A similar effect on the baroreflex has been described previously [35]. This confirms that the sites identified here are the source of cardiac vagal outflow and that the majority of CVPN could be targeted with two bilateral microinjections.

### Role of ionotropic GABA_A _and strychnine sensitive glycine receptors in areas of the medulla containing CVPN in the tonic and baroreflex control of HR

**Figure 2:**
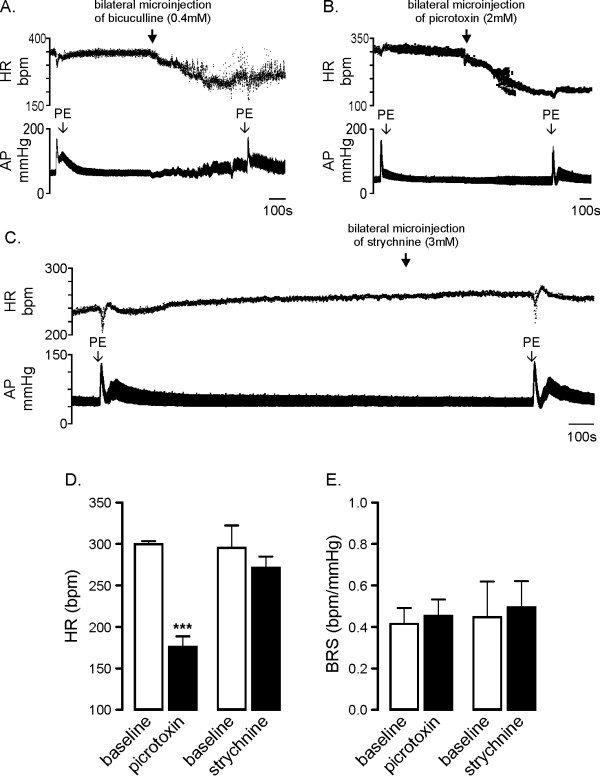
**Role of ionotropic GABA_A _and strychnine sensitive glycine receptors in areas of the medulla containing cardiac vagal preganglionic neurons in the tonic and baroreflex control of HR**. Panel A and B show the effects of bilateral microinjection of the GABA_A _antagonists bicuculline (0.4 mM, n = 4) and picrotoxin (2 mM, n = 6) respectively on resting heart rate (HR) and baroreflex evoked bradycardia induced using phenylephrine (PE). Both bicuculline and picrotoxin evoked large and similar decreases in resting HR. Bicuculline, however, evoked arrhythmia and the effects on baroreflex bradycardia could not be quantified. Panel C shows the effects of bilateral microinjection of strychnine (3 mM, n = 4) on resting HR and baroreflex bradycardia. Group data (panels D and E) shows that picrotoxin evoked a large decrease in resting HR whereas strychnine had no effect (panel D). Neither picrotoxin nor strychnine affected baroreflex bradycardia or baroreflex sensitivity (BRS, panel E). *** *P *< 0.001.

The effects of bilateral microinjections of the GABA_A _antagonist picrotoxin on HR and baroreflex function are shown in Figure 2A. Bilateral picrotoxin elicited a profound reduction in HR (from 300 ± 4 bpm to 176 ± 13 bpm, *P *< 0.01, Figure 2D); however, in contrast to activation of GABA_A _receptors (Figure 1D), blockade of GABA_A _receptors did not alter BRS (Figure 2E). Bilateral microinjection of bicuculline, another GABA_A _antagonist, as illustrated in Figure 2B, also evoked a profound decrease in HR (335 ± 11 v 185 ± 30 bpm, control v bicuculline *P *< 0.01 n = 4) decreasing HR to a level similar to that seen following picrotoxin (*P *= 0.76). However bicuculline consistently evoked arrhythmias possibly resulting from the blockade of after-hyperpolarisation potentials (I_AHP_) arising due to the blockade of SK channels [36]. As a result BRS could not be analysed.

The effect of bilateral microinjection of strychnine on HR and baroreflex function is shown in Figure 2C. Inhibition of strychnine-sensitive glycine receptors had no effect on resting HR (296 ± 27 bpm v 272 ± 13 bpm *P *= 0.2051 Figure 2D) or BRS (0.44 ± 0.17 bpm/mmHg v 0.49 ± 0.13 bpm/mmHg, *P *= 0.60 Figure 2E).

### Role of ionotropic glutamate receptors in areas of the medulla containing CVPN in the tonic and baroreflex control of HR

**Figure 3:**
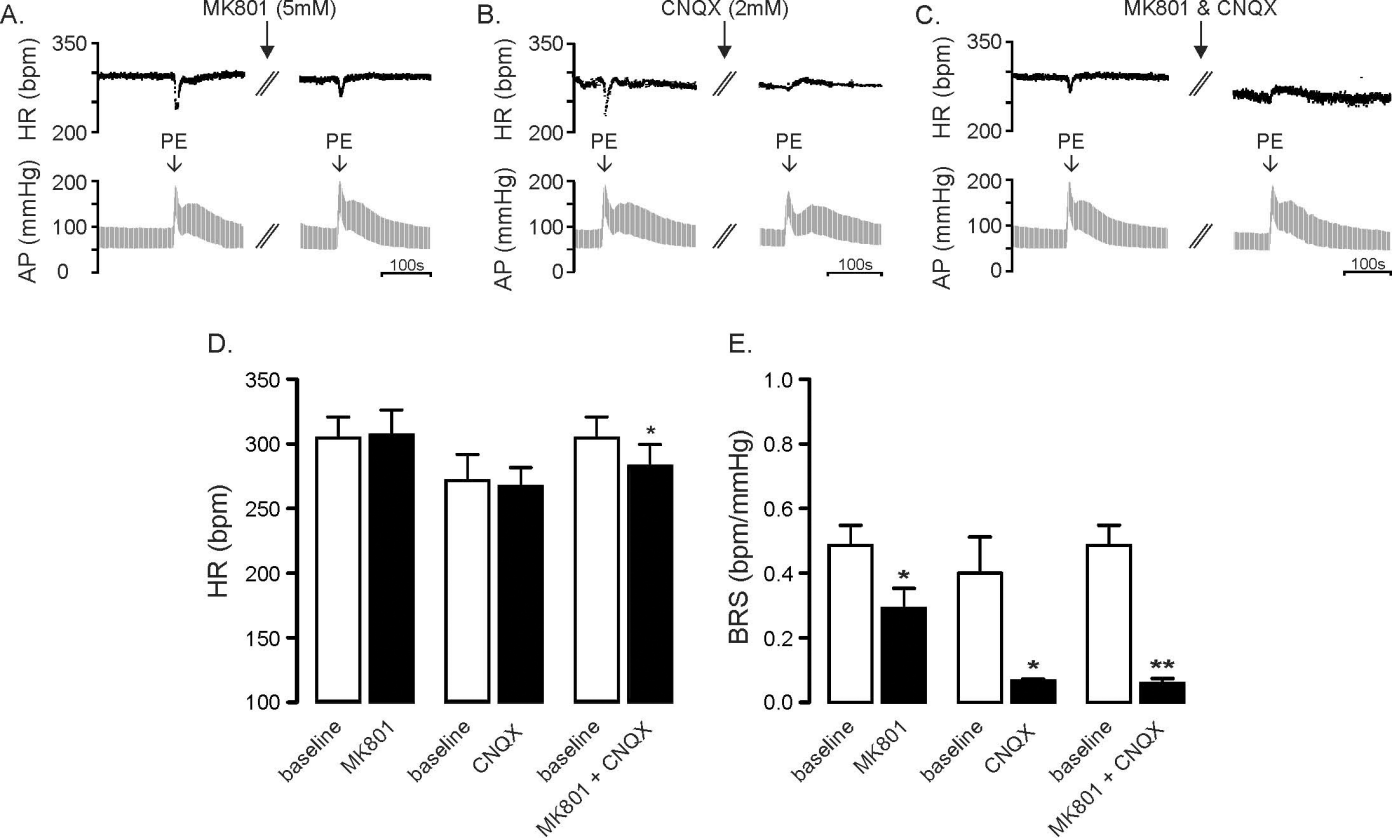
**Role of ionotropic glutamate receptors in areas of the medulla containing CVPN in the tonic and baroreflex control of HR**. Panel A illustrates the effects of bilateral microinjection of the NMDA antagonist MK801 (5 mM, n = 4), panel B illustrates the effects of bilateral microinjection of the AMPA antagonist CNQX (2 mM, n = 3) and panel C illustrates the effects of bilateral microinjection of CNQX following MK801 (n = 4) on resting heart rate (HR) and baroreflex bradycardia. Panel D illustrates the effects of these antagonists on resting HR. Neither MK801 nor CNQX alone altered resting HR; however, combined microinjection of these antagonists evoked a small but significant decrease in resting HR. In panel E the effect of these antagonists on baroreflex function are shown. Microinjection of MK801 evoked a modest decrease in baroreflex sensitivity (BRS) whereas microinjection of CNQX dramatically reduced BRS and abolished all baroreflex function. Combined microinjection of MK801 and CNQX reduced BRS to levels similar to that observed following microinjection of CNQX alone. * *P *< 0.05, ** *P *< 0.01.

The effects of bilateral inhibition of NMDA or AMPA receptors in medullary regions containing CVPN on HR and baroreflex function are shown in Figure 3A and 3B respectively. Blockade of NMDA receptors with MK801 or AMPA receptors with CNQX did not change resting HR (Figure 3D). BRS, however, was reduced by approximately 40% in response to blockade of NMDA receptors (0.49 ± 0.06 v 0.29 ± 0.06 bpm/mmHg, *P *< 0.05 Figure 3E) and 83% in response to blockade of AMPA receptors (0.40 ± 0.11 v 0.07 ± 0.01 bpm/mmHg, *P *< 0.05 Figure 3E).

The effects of combined AMPA/NMDA receptor blockade in the region containing CVPN on HR and baroreflex function are shown in Figure 3C. A small reduction in resting HR (306 ± 16 bpm v 284 ± 17 bpm *P *< 0.05, Figure 3D) was observed and BRS was reduced by approximately 85% (0.40 ± 0.11 v 0.06 ± 0.01 bpm/mmHg, *P *< 0.05 Figure 3E). BRS following combined AMPA/NMDA receptor blockade was similar to that following AMPA receptor blockade alone (*P *= 0.33).

### Role of GABAergic and glycinergic neurotransmission in regions of the medulla containing CVPN in cardio-vagal responses to 5-HT_1A _receptor activation

**Figure 4:**
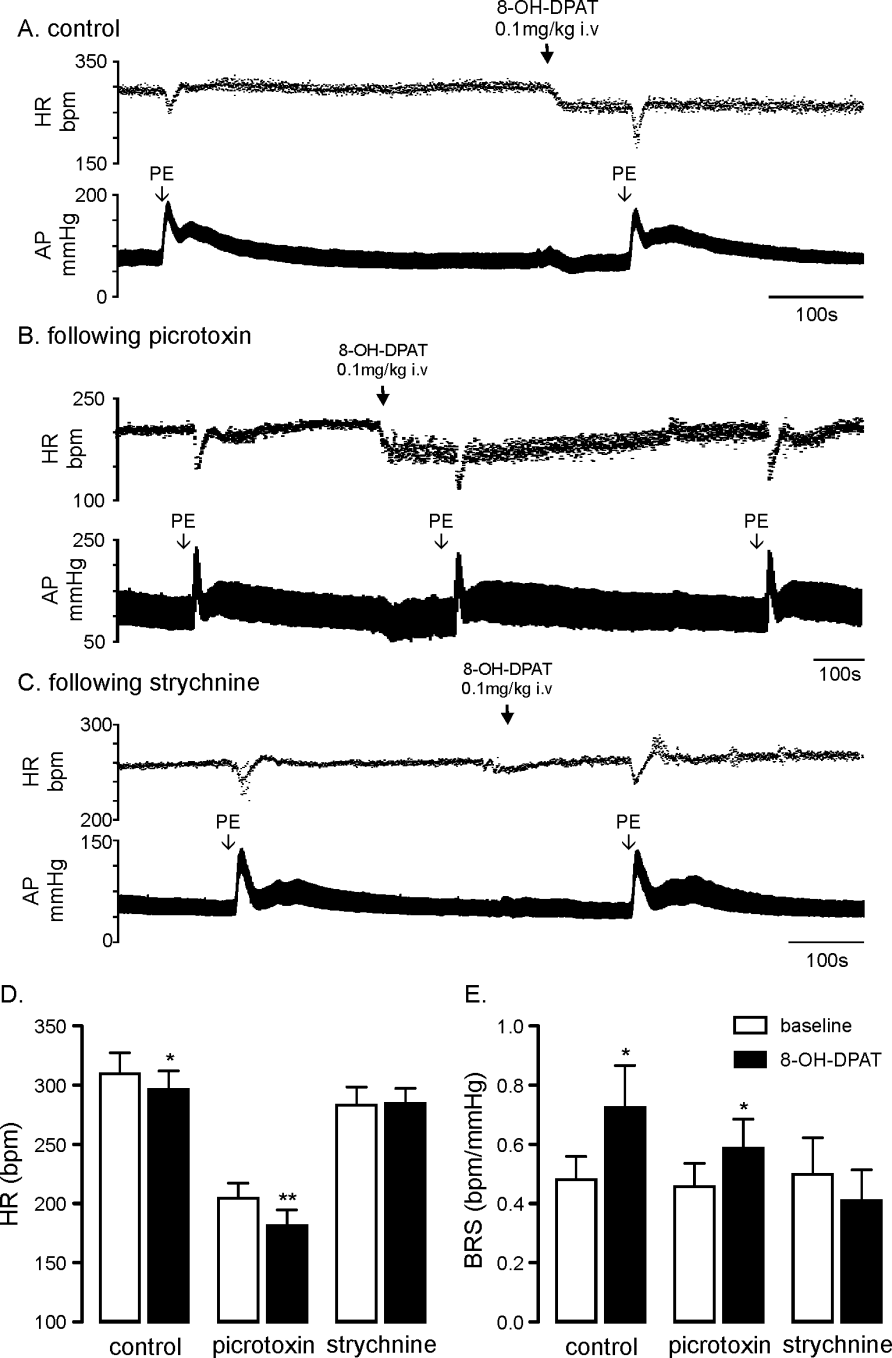
**Role of GABAergic and glycinergic neurotransmission in regions of the medulla containing cardiac vagal preganglionic neurons in cardio-vagal responses to 5-HT_1A _receptor activation**. Panel A illustrates the effects of intravenous injection of the 5-HT_1A _agonist 8-OH-DPAT (0.1 mg/kg, n = 9) on heart rate (HR) and baroreflex bradycardia. Panels B and C shows the intravenous injection of 8-OH-DPAT on HR and baroreflex bradycardia following prior microinjection of the GABA_A _antagonist picrotoxin (2 mM, n = 6) and glycine antagonist strychnine (3 mM, n = 4) into regions of the medulla containing cardiac vagal preganglionic neurons respectively. Panel D shows that an injection of 8-OH-DPAT alone evokes a small bradycardia which is not altered by prior microinjection of picrotoxin. Prior microinjection of strychnine prevents this bradycardia. Panel E shows that injections of 8-OH-DPAT alone increase baroreflex sensitivity (BRS). This increase in BRS is still seen following prior microinjection of picrotoxin but not strychnine. * *P *< 0.05, ** *P *< 0.01.

The effect of intravenous administration of 8-OH-DPAT (0.1 mg/kg) on HR and baroreflex function is illustrated in Figure 4A. Activation of 5-HT_1A _receptors with 8-OH-DPAT evoked a small but significant decrease in resting HR (from 310 ± 17 bpm control to 296 ± 15 bpm, *P *< 0.05 Figure 4D) and a 40% increase in BRS (from 0.49 ± 0.07 bpm/mmHg to 0.73 ± 0.14 bpm/mmHg, *P *< 0.05 Figure 4E) as previously described [23].

The effect of intravenous 8-OH-DPAT on HR and baroreflex function following microinjection of picrotoxin or strychnine is shown in Figure 4B and 4C respectively. Following microinjection of picrotoxin, intravenous administration of 8-OH-DPAT still decreased resting HR (from 205 ± 13 bpm to 181 ± 14 bpm, *P *< 0.01 Figure 4D) and increased BRS (from 0.45 ± 0.08 bpm/mmHg to 0.59 ± 0.09 bpm/mmHg, *P *< 0.05 Figure 4E). Following microinjection of strychnine, intravenous administration of 8-OH-DPAT (0.1 mg/kg) no longer evoked a decrease in HR (283 ± 15 bpm v 285 ± 13 bpm *P *= 0.75 Figure 4D) or an increase in BRS (0.49 ± 0.13 bpm/mmHg v 0.41 ± 0.10 bpm/mmHg, *P *= 0.29 Figure 4E). The inhibitory actions of 8-OH-DPAT on HR were unaffected by prior microinjection of picrotoxin but abolished through prior microinjection of strychnine (-13 ± 5 bpm vs. -23 ± 6 bpm vs. 2 ± 5 bpm control vs. picrotoxin vs. strychnine *P *< 0.05).

## Discussion

The major findings of the present study are that in regions of the brain containing CVPN: 1. Activation of GABA_A _receptors is required to set the resting level of HR; 2. Baroreflex mediated bradycardia is dependent upon activation of AMPA receptors to a greater extent than NMDA receptors 3. Strychnine sensitive glycine receptors are not involved in the tonic or reflex control of HR but their activation is required to enable the bradycardiac and baroreflex facilitating effects of 5-HT_1A _receptor activation.

### Role of ionotropic GABA and glycine receptors in regions of the medulla containing CVPN in the tonic and reflex control of HR

We have shown that GABA_A _receptors, but not strychnine sensitive glycine receptors, are required for setting the level of HR. That GABA_A _receptors are vital for setting the tonic level of vagal outflow to the heart is well established and our findings support the notion that CVPN receive a substantial GABAergic input [12,35]. The bradycardia evoked following bilateral microinjection of either picrotoxin or bicuculline is likely due to blockade of postsynaptic GABA_A _receptors on CVPN as focal application of picrotoxin blocks inhibitory postsynaptic currents in CVPN [13,37]. Our finding that bilateral microinjection of strychnine does not alter resting HR is in contrast with previous reports where unilateral microinjection of strychnine into the NA decreased resting HR suggesting a role for strychnine sensitive glycine receptors in the control of HR at least in cats [12]. Nevertheless, in rats, microinjection of strychnine does not affect the tachycardic response to microinjection of glycine in cardioinhibitory sites of the medulla [14]. Together with our results this suggests that strychnine-sensitive glycine receptors are not involved in the tonic control of cardiac vagal outflow in the rat.

While the role of GABA_A _and strychnine sensitive glycine receptors in the tonic control of HR has been investigated previously, how these receptors are involved in evoking reflex changes in HR has not. It has been hypothesised that disinhibiting CVPN through inhibition of GABAergic or glycinergic inputs to CVPN can improve BRS through the facilitation of excitatory inputs to CVPN [23,38,39]. We tested this and showed that bilateral microinjection of picrotoxin or strychnine did not alter BRS. This demonstrates for the first time that neither GABA_A _nor strychnine sensitive glycine receptors are required for the baroreflex activation of cardiac vagal outflow, at least in regions of the medulla containing CVPN.

### Role of ionotropic glutamate receptors in regions of the medulla containing CVPN in the tonic and reflex control of HR

Activation of ionotropic glutamate receptors, which are found on the soma and dendrites of CVPN [40], with L- glutamate produces a decrease in HR (as seen here to identify CVPN and previously [18,20,34]). However, combined blockade of NMDA and AMPA receptors did not evoke an expected increase in HR, but paradoxically a bradycardia. This confirms the study of Guyenet et al [15] who observed bradycardia following microinjection of kynurenic acid into the region of the NA. We extended these findings by showing that blockade of either NMDA or AMPA receptors alone did not evoke any change in HR suggesting that there is not a substantial tonic ionotropic glutamatergic input to CVPN. In light of the small bradycardia evoked by combined receptor blockade, it is possible that there is some tonic glutamatergic control of inhibitory inputs to CVPN. Glutamatergic modulation, involving both NMDA and AMPA receptors, of GABAergic inputs to CVPN in the DMNV but not NA has been described previously [41]. It remains possible that glutamatergic regulation of glycinergic inputs may exist in the NA.

Baroreflex mediated changes in parasympathetic outflow to the heart occur via glutamatergic inputs from the nucleus tractus solitarius (NTS) to cardiac vagal preganglionic neurons (CVPN) located principally in the nucleus ambiguus (NA). Several lines of evidence support this: Stimulation of the baroreflex *in vivo *evokes a reflex increase in CVPN activity which correlates with the reflex bradycardia evoked [7]; Microinjection kynurenic acid, a non-specific glutamate antagonist, into the region of the NA prevents vagally mediated baroreflex bradycardia [15]; Electrical stimulation of depressor sites within the NTS increases CVPN unit activity *in vivo *[17]; Stimulation of the NTS *in vitro *evokes excitatory postsynaptic currents in CVPN which are inhibited by combined blockade of NMDA and AMPA receptors [16].

While there is substantial evidence to indicate that glutamatergic inputs to CVPN are necessary for the generation of baroreflex bradycardia, the receptor subtype(s) responsible for eliciting these reflex changes is unknown. Both NMDA and AMPA receptors are located on the soma and dendrites of CVPN [40]. In the present study, inhibition of NMDA receptors modestly attenuated BRS whereas blockade of AMPA receptors completely abolished baroreflex control of HR. The reduction in BRS evoked by CNQX alone was similar to that following combined NMDA/AMPA receptor blockade. Thus, the integrity of AMPA receptors within CVPN alone is critical to the baroreflex activation of cardiac vagal outflow whereas NMDA receptors play a more minor role. This contrasts with the responses obtained in other vagal motoneurons in the NA, where NMDA receptors mediate the effects evoked by stimulation of the NTS [42].

Our findings also contrast with those some of those described within other medullary regions of the baroreflex arc. In the NTS some report that baroreflex neurotransmission is dependent upon NMDA receptor activation [43] and others, non-NMDA receptor activation [44]. Nevertheless, the majority report that both receptor subtypes are required for full expression of the baroreceptor reflex [45-49]. The caudal ventrolateral medulla, which also receives baroreceptor input from the NTS, is excited by activation of both AMPA and NMDA receptors [50]. A greater dependence on AMPA over NMDA receptors for the full expression of baroreceptor mediated bradycardia therefore appears to be unique to the vagal outflow of the baroreflex.

### Role of ionotropic GABAergic and glycinergic neurotransmission within regions of the medulla containing CVPN in the cardio-vagal responses to 5-HT_1A _receptor activation

We have confirmed previous findings which demonstrate that systemic administration of 8-OH-DPAT results in a vagally mediated bradycardia and potentiation in baroreflex mediated bradycardia [22,23]. We have then extended these findings to determine the central mechanisms responsible for this 8-OH-DPAT evoked potentiation in reflex bradycardia.

8-OH-DPAT is an agonist at both 5-HT_1A _and 5-HT_7 _receptors [51], although the actions at the 5-HT_1A _receptor are presumed responsible for the potentiation in reflex vagal outflow. Kellett and others [24] demonstrated that intracisternal administration of WAY-100635, the 5-HT_1A _selective antagonist, attenuates baroreflex mediated bradycardia indicating that 5-HT_1A _receptors in the medulla are responsible for the facilitation of baroreflex bradycardia evoked by 8-OH-DPAT. Wang and Ramage [25] further showed that iontophoresis of WAY-100635 onto CVPN attenuates the increase in activity of these neurons in response to stimulation of pulmonary C fibres.

The most logical explanation for the 8-OH-DPAT mediated facilitation of baroreflex bradycardia is through inhibition of GABAergic or glycinergic inputs to CVPN. In support of this hypothesis, in slice preparation, application of 8-OH-DPAT inhibits both GABAergic and glycinergic inputs to CVPN [26,27]. To confirm the physiological relevance of these findings we blocked GABA_A _receptors or strychnine-sensitive glycine receptor in the vicinity of CVPN and examined the response of systemic 8-OH-DPAT on the tonic and reflex bradycardia evoked. We found that intravenous 8-OH-DPAT administration evoked bradycardia and potentiated BRS in the presence of GABA_A _receptor blockade whereas 8-OH-DPAT failed to evoke a response when strychnine sensitive glycine receptors were antagonised. It is unlikely that the ability of 8-OH-DPAT to improve BRS in the presence of picrotoxin is related to the HR lowering effects of picrotoxin. An inverse relationship does exist between resting HR and BRS, such that the lower the HR the greater the BRS [52]; however, if the ability for 8-OH-DPAT to improve BRS in the presence of picrotoxin was solely due to a HR lowering effect of picrotoxin then BRS should have improved following microinjection of picrotoxin. As this was not the case, this indicates that 8-OH-DPAT improves BRS independent of the resting level of HR. Furthermore, our findings indicate that the bradycardiac and baroreflex enhancing effects of 8-OH-DPAT are dependent upon functional glycinergic neurotransmission in the vicinity of CVPN.

The finding that 8-OH-DPAT acts via a glycinergic mechanism was surprising as microinjection of strychnine did not affect HR or BRS. As 5-HT_1A _receptors are purported to be tonically involved in the reflex activation of CVPN [22,24], we expected that if 8-OH-DPAT were acting on glycinergic neurons directly innervating CVPN then strychnine would mimic the effects of 8-OH-DPAT. While we cannot reconcile this perplexing finding, a previous *in vitro *study indicated that glycinergic inputs to CVPN are more sensitive to 8-OH-DPAT than GABAergic inputs providing some support for our findings. The location of the 5-HT_1A _receptor with respect to both CVPN and glycinergic input is unknown. Our current hypothesis is that the 5-HT_1A _receptor is located upstream of a glycinergic input to CVPN.

## Conclusions

Diseases including hypertension, depression and kidney failure are associated with impaired function of vagal inputs to the heart [53-55]. Understanding how neurochemicals control cardiac vagal function is essential to determining what can go wrong in disease. Until now central neurochemical inputs to CVPN responsible for the tonic and reflex control of HR have been poorly investigated. Here we present a comprehensive investigation of the role of ionotropic GABAergic, glycinergic and glutamatergic inputs in the tonic and reflex control of HR. We also provide a mechanism by which activation of 5-HT_1A _receptors can improve vagal outflow to the heart. This is an important contribution as activation of 5-HT_1A _receptors does not improve baroreflex function in rodent models of depression suggesting that the interaction between 5-HT_1A _receptors and glycinergic neurons may be involved in the aetiology of abnormal vagal control of HR in depression [23]. This study enhances our understanding of neural control of the heart and will provide a reference point for future studies examining central mechanisms responsible for impaired cardiac vagal control in disease.

## Authors' contributions

CMH carried out the experiments, analysed the data and drafted the manuscript. AKG conceived, designed and coordinated the study and critically revised the manuscript. Both authors read and approved the final manuscript.
